# A Question of Dose? Ultra-Low Dose Chest CT on Photon-Counting CT in People with Cystic Fibrosis

**DOI:** 10.3390/tomography11120134

**Published:** 2025-11-27

**Authors:** Marcel Opitz, Matthias Welsner, Halil I. Tazeoglu, Florian Stehling, Sivagurunathan Sutharsan, Dirk Westhölter, Erik Büscher, Christian Taube, Nika Guberina, Denise Bos, Marcel Drews, Daniel Rosok, Sebastian Zensen, Johannes Haubold, Lale Umutlu, Michael Forsting, Marko Frings

**Affiliations:** 1 Institute of Diagnostic and Interventional Radiology and Neuroradiology, University Hospital Essen, Hufelandstraße 55, 45147 Essen, Germany; marcel.opitz@uk-essen.de (M.O.); halilibrahim.tazeoglu@uk-essen.de (H.I.T.); nika.guberina@uk-essen.de (N.G.); denise.bos@uk-essen.de (D.B.); marcel.drews@uk-essen.de (M.D.); daniel.rosok@uk-essen.de (D.R.); sebastian.zensen@uk-essen.de (S.Z.); johannes.haubold@uk-essen.de (J.H.); lale.umutlu@uk-essen.de (L.U.); michael.forsting@uk-essen.de (M.F.); 2 Department of Pulmonary Medicine, University Hospital Essen-Ruhrlandklinik, Adult Cystic Fibrosis Center, University Duisburg-Essen, Tüschener Weg 40, 45239 Essen, Germany; matthias.welsner@rlk.uk-essen.de (M.W.); sivagurunathan.sutharsan@rlk.uk-essen.de (S.S.); dirk.westhoelter@rlk.uk-essen.de (D.W.); christian.taube@rlk.uk-essen.de (C.T.); 3 Pediatric Pulmonology and Sleep Medicine, Cystic Fibrosis Center, Children’s Hospital, University Duisburg-Essen, Hufelandstraße 55, 45147 Essen, Germany; florian.stehling@uk-essen.de; 4 Department of Radiotherapy, University Medicine Essen, University Duisburg-Essen, 45147 Essen, Germany

**Keywords:** cystic fibrosis, adults, photon-counting chest CT, radiation dosage, ultra-low-dose

## Abstract

**Objective:** Chest computed tomography (CT) is a key component of the diagnostic assessment of people with cystic fibrosis (PwCF) and is increasingly replacing chest radiography. Due to improvements in life expectancy, radiation exposure has become a growing concern in PwCF. Photon-counting CT (PCCT) has the potential to reduce the risk of radiation-induced malignancies while maintaining diagnostic accuracy. This study aimed to compare the radiation dose and image quality of low-dose high-resolution (LD-HR) and ultra-low-dose high-resolution (ULD-HR) CT protocols using PCCT in PwCF. **Methods:** This retrospective study included 72 PwCF, with 36 undergoing a LD-HR chest CT protocol and 36 receiving an ULD-HR protocol on a PCCT. The radiation dose and image quality were assessed by comparing the effective dose and signal-to-noise ratio (SNR). Three blinded radiologists evaluated the overall image quality, sharpness, noise, and assessability of the bronchi, bronchial wall thickening, and bronchiolitis using a five-point Likert scale. **Results:** The ULD-HR PCCT protocol reduced radiation exposure by approximately 65% compared with the LD-HR PCCT protocol (median effective dose: 0.19 vs. 0.55 mSv, *p* < 0.001). While LD-HR images were consistently rated higher than ULD-HR images (*p* < 0.001), both protocols maintained diagnostic significance (median image quality rating of “4-good”). The average SNR of the lung parenchyma was significantly lower with ULD-HR PCCT compared to LD-HR PCCT (*p* < 0.001). **Conclusions:** ULD-HR PCCT significantly reduced radiation exposure while maintaining good diagnostic image quality in PwCF. The effective dose of ULD-HR PCCT is only twice that of a two-plane chest X-ray, making it a viable low-radiation alternative for routine imaging in PwCF.

## Simple Summary

Chest CT scans are important for monitoring lung problems in people with cystic fibrosis (CF). However, repeated scans can expose patients to radiation, which is a concern as people with CF are now living longer due to improved treatment options. A newer type of CT technology, called photon-counting CT (PCCT), may help reduce this radiation. In this study, researchers compared two PCCT settings (low-dose and ultra-low-dose) in 72 people with CF. The results showed that the ultra-low-dose PCCT scans used about 65% less radiation than the low-dose version. Even though the image quality was slightly lower, doctors still rated the ultra-low-dose version as good enough for diagnosis. In fact, the radiation from the ultra-low-dose scan was only about twice that of a standard two-view chest X-ray, which is very low for a CT scan.

## 1. Introduction

Radiation exposure in people with cystic fibrosis (PwCF) is an increasing concern because of their improved life expectancy [1,2,3]. In clinical practice, both chest X-ray and computed tomography (CT) are commonly used for routine follow-up in PwCF [4,5]. However, chest CT surpasses both chest X-ray and pulmonary function tests in accurately assessing disease severity and detecting early CF-related lung abnormalities, such as bronchiectasis, mucus plugging, and air trapping [6,7]. Notably, CT scans can often identify pathological changes before symptoms appear or abnormalities become visible on chest X-ray. This superior sensitivity is crucial for enabling timely treatment and potentially slowing disease progression. The slowing of disease progression and the associated increase in life expectancy have gained renewed importance in recent years, particularly due to the introduction of cystic fibrosis transmembrane conductance regulator (CFTR) modulator therapy with elexacaftor/tezacaftor/ivacaftor [8,9,10]. In line with these findings, several studies have highlighted a significant increase in CT utilization in PwCF, leading to a high cumulative lifetime radiation dose [1,3,11].

In recent years, photon-counting CT (PCCT) has emerged as an innovative technology with proven benefits in pulmonary imaging. Compared to conventional energy-integrating detector CT (EID-CT) systems, PCCT allows for a substantial reduction in radiation dose in low-dose chest CT protocols while simultaneously providing superior image quality [12,13]. Moreover, research indicates that with ultra-low-dose CT (ULD-CT) using PCCT, radiation exposure can be reduced to levels comparable to chest X-rays while offering greater clinical impact owing to improved diagnostic accuracy [14].

A European guidance recommends that ULD CT scans in children and adults with cystic fibrosis (CF) should be performed at an effective dose of 0.08 mSv [5]. This refers to a study investigating the effect of ivacaftor in 33 PwCF, in which effective dose values with a ULD of 0.08 mSv were achieved on EID-CT. However, the study did not explicitly evaluate the image quality of the CT examinations [15].

Following the ALARA (As Low As Reasonably Achievable) principle, our goal was to establish a chest PCCT protocol for PwCF that maintains diagnostic accuracy while minimizing radiation exposure. We hypothesized that ultra-low-dose high-resolution (ULD-HR) PCCT would provide good diagnostic image quality comparable to low-dose high-resolution (LD-HR) PCCT while substantially reducing radiation dose.

## 2. Materials and Methods

### 2.1. Study Population and Ethics Statement

This single-center retrospective study included 72 PwCF from two cohorts who underwent chest CT imaging as part of their routine clinical care between July 2023 and July 2024. The first cohort of 36 PwCF underwent routine LD-HR PCCT scans between July and November 2023, while another cohort of 36 PwCF underwent routine PCCT scans using a ULD-HR protocol between November 2023 and July 2024 (NAEOTOM Alpha, Siemens Healthineers, Erlangen, Germany). An interindividual comparison of image quality and radiation dose was performed without any preselection based on factors such as age, sex, weight, or other characteristics. This study was conducted in accordance with the guidelines of the Declaration of Helsinki and was approved by the Institutional Review Board (23-11602-BO, Ethics Committee of the Medical Faculty of the University of Duisburg-Essen). Informed consent was waived due to the retrospective nature of the study. All imaging procedures were conducted in accordance with standard clinical protocols for diagnostic purposes, and patient data were anonymized before being included in the study.

### 2.2. CT Protocols and Image Acquisition

The scan parameters for both protocols were as follows: tube voltage of 100 kVp with a tin filter, detector configuration of 144 × 0.4 mm, automatic tube current and voltage modulation (tube current: CARE Dose4D and tube voltage: CARE kV, both Siemens Healthineers, Erlangen, Germany), and spiral pitch factor of 1.5. In newer Siemens CT scanners, the IQ level is used to modulate the tube current. A higher IQ level corresponds to an increased tube current. An image quality level of 15 was selected for the LD-HR protocol, and an image quality level of 5 was selected for the ULD-HR protocol. In both protocols, images were reconstructed for lung tissue using a BI64 convolution kernel (window settings: center −350, width 1500) with a slice thickness of 1 mm in axial slices. Scans for both protocols were reconstructed using a 1024 × 1024 matrix with quantum-iterative reconstruction. The technologist manually adjusted the field of view based on the patient’s size in the scout view. All CT scans were performed during breath-hold at full inspiration. Patients were positioned supine with their arms elevated above their heads and an isocentric configuration.

### 2.3. Quantitative Image Quality Analysis

Regions of interest were manually placed as circles with an area of 1 mm^2^ at consistent locations (carina level) for each patient within the healthy lung parenchyma, autochthonous back muscles, and air outside the patient. This ROI size was chosen in order to identify comparable findings in all patients and because some pathologies were very extensive. Measurements were performed on a single slice of the sample. The signal within each region of interest was measured in Hounsfield units, and the image noise was calculated as the standard deviation of the region of interest. The signal-to-noise ratio was determined by dividing the Hounsfield units of the region of interest by its corresponding standard deviation.

### 2.4. Qualitative Image Quality Analysis

Three blinded radiologists with 5 (M.F.), 6 (H.T.), and 7 (M.O.) years of experience in chest CT independently assessed the overall image quality, image sharpness, image noise, and the assessability of bronchi, bronchial wall thickening, and bronchiolitis of the CT scans from 72 PwCF using a 5-point Likert scale. The scale for image quality, sharpness, and assessability of the bronchi, bronchial wall thickening, and bronchiolitis was defined as follows: 1, insufficient; 2, sufficient; 3, satisfactory; 4, good; and 5, very good. For image noise, the scale was as follows: 1, very high; 2, high; 3, moderate; 4, low; and 5, very low. The assessability of bronchi, bronchial wall thickening, and bronchiolitis were selected as an evaluation criterion to ensure that CF-relevant airway pathologies were appropriately captured in the image analysis. The CT scans were anonymized and blinded before being presented to the readers. The images were displayed using the Centricity™ Universal Viewer PACS (GE Healthcare, Düsseldorf, Germany) with multiplanar reformation.

### 2.5. Radiation Dose

The radiation dose was assessed using an automated dose monitoring software (Radimetrics Enterprise Platform, Bayer Healthcare, Leverkusen, Germany). This software extracts radiation exposure data and patient demographic information from the Digital Imaging and Communications in Medicine headers stored in the picture archiving and communication system [16]. The evaluation included the volumetric CT dose index, dose-length product, and effective dose. To estimate cancer risk for specific organs, organ doses were calculated for the lungs, heart, skin, bones, esophagus, and thymus. The software utilized Monte Carlo simulations to compute both the effective radiation and organ doses, applying the weighting factors outlined in the International Commission on Radiological Protection publication 103 [17,18]. The tissue weighting factor is used to account for the radiation sensitivity of an organ or tissue and to determine its relative contribution to the effective dose [19].

### 2.6. Statistical Analysis

Statistical analyses were conducted using RStudio (Version 2023.12.0). Data following a normal distribution are reported as mean ± standard deviation, while non-normally distributed data are presented as median with interquartile range (IQR). The Kolmogorov–Smirnov test was used to assess the normal distribution of the data. For normally distributed data, a paired *t*-test was applied, and for non-normally distributed data, the Wilcoxon test was used. A two-sided test design was used for statistical analyses of age and BMI. All other parameters were evaluated using a one-sided hypothesis test, where the null hypothesis assumed no difference between LD and ULD, and the alternative hypothesis assumed that ULD was either superior or inferior to the given parameter. A two-sided test did not yield different results. The Chi-square test was used for the categorical variable ‘gender.’ Inter-rater reliability was evaluated using Fleiss’ Kappa, interpreted as follows: <0.00, poor agreement; 0.00–0.20, slight agreement; 0.21–0.40, fair agreement; 0.41–0.60, moderate agreement; 0.61–0.80, substantial agreement; and ≥0.81, almost perfect agreement [20]. Statistical significance was defined as *p* < 0.05.

## 3. Results

### 3.1. Study Population

The LD-HR cohort comprised 10 women (28%) and 26 men (72%) with a mean age of 35.89 ± 12.48 years and BMI of 21.83 ± 2.99 kg/m^2^ (Table 1). The ULD-HR cohort included 20 women (56%) and 16 men (44%) with a mean age of 33.19 ± 10.66 years and BMI of 23.08 ± 4.66 kg/m^2^ (Table 1). There were no statistically significant differences between the groups in terms of age, sex, and BMI (all *p* > 0.05).

### 3.2. Quantitative Image Quality Analysis

**Figure 1:**
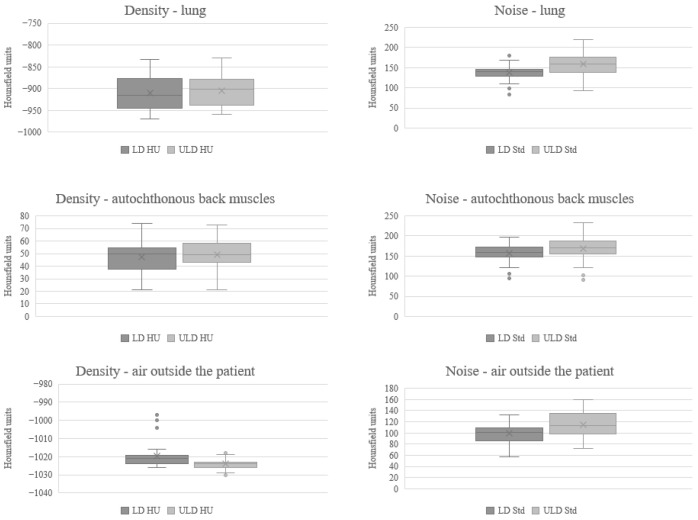
Comparison of Mean and Standard Deviation in Quantitative Image Quality Analysis. HU Hounsfield units, LD-HR low-dose high-resolution, Std standard deviation, ULD-HR ultra-low-dose high-resolution.

The signals were similar for the lungs, autochthonous back muscles, and air outside the patient, whereas the absolute value of the noise on the ULD-HR scans was higher than that on the LD-HR (SD LD-HR lung: 137.97, autochthonous back muscles: 157.64, air outside the patient: 99.33, SD ULD-HR: lung: 158.86, autochthonous back muscles: 169.50, air outside the patient: 114.92, Table 2 and Figure 1). The magnitude of signal-to-noise ratio was significantly lower on ULD-HR scans for the lung and air outside the patient (LD-HR lung: 6.75, ULD-HR lung: 5.88, *p* < 0.001; LD-HR air outside the patient: 10.58, ULD-HR air outside the patient: 9.30, *p* = 0.012, Table 2). There was no statistically significant difference in the signal-to-noise ratio of the autochthonous back muscles (*p* = 0.322, Table 2). Due to the negative signal values in the lungs and air outside the patient, the signal-to-noise ratio was presented as magnitude.

### 3.3. Qualitative Image Quality Analysis

**Figure 2:**
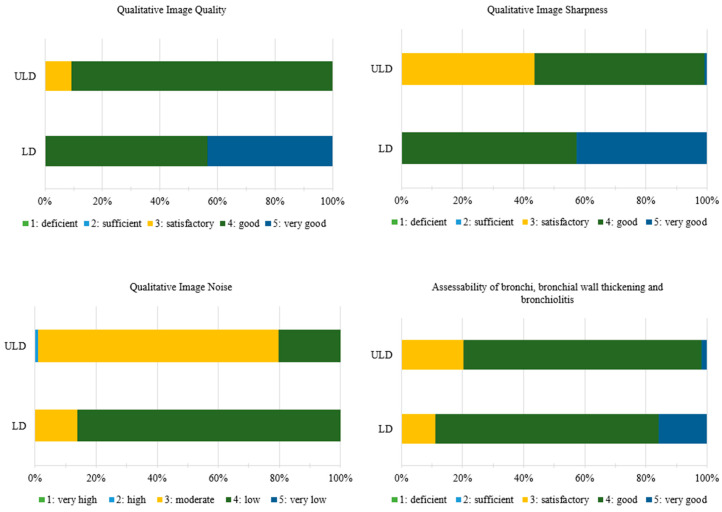
Ratings of Qualitative Image Quality Analysis. Medians for qualitative image quality, image sharpness and assessability of the bronchi, bronchial wall thickening, and bronchiolitis are identical and distributional shifts account for significance. ULD: n = 36, LD: n = 36. LD-HR low-dose high-resolution, ULD-HR ultra-low-dose high-resolution.

Overall, the ULD-HR images received slightly lower ratings than the LD-HR images. Because the medians are often identical, it is important to refer to Figure 2 to fully understand and interpret the results. Since the results of qualitative image quality analysis were not normally distributed, a Wilcoxon test was used to evaluate the results. The LD-HR images were rated significantly higher in terms of image quality (LD-HR: 4 [IQR: 1], ULD-HR: 4 [IQR: 0], *p* < 0.001, Figure 2 and Table 3), image sharpness (LD-HR: 4 [IQR: 1], ULD-HR: 4 [IQR: 1], *p* < 0.001, Figure 2 and Table 3), and assessability of the bronchi, bronchial wall thickening, and bronchiolitis (LD-HR: 4 [IQR: 1], ULD-HR: 4 [IQR: 0], *p* < 0.001, Figure 2 and Table 3). Image noise was rated significantly higher in ULD-HR images than in LD-HR images (LD-HR: 4 [IQR: 0]; ULD-HR: 3 [IQR: 0]; *p* < 0.001, Figure 2 and Table 3).

The interrater reliability for both the LD-HR and ULD-HR protocols indicated at least substantial agreement, with slightly higher values observed for the LD-HR protocol than for the ULD-HR protocol (Table 3).

**Figure 3:**
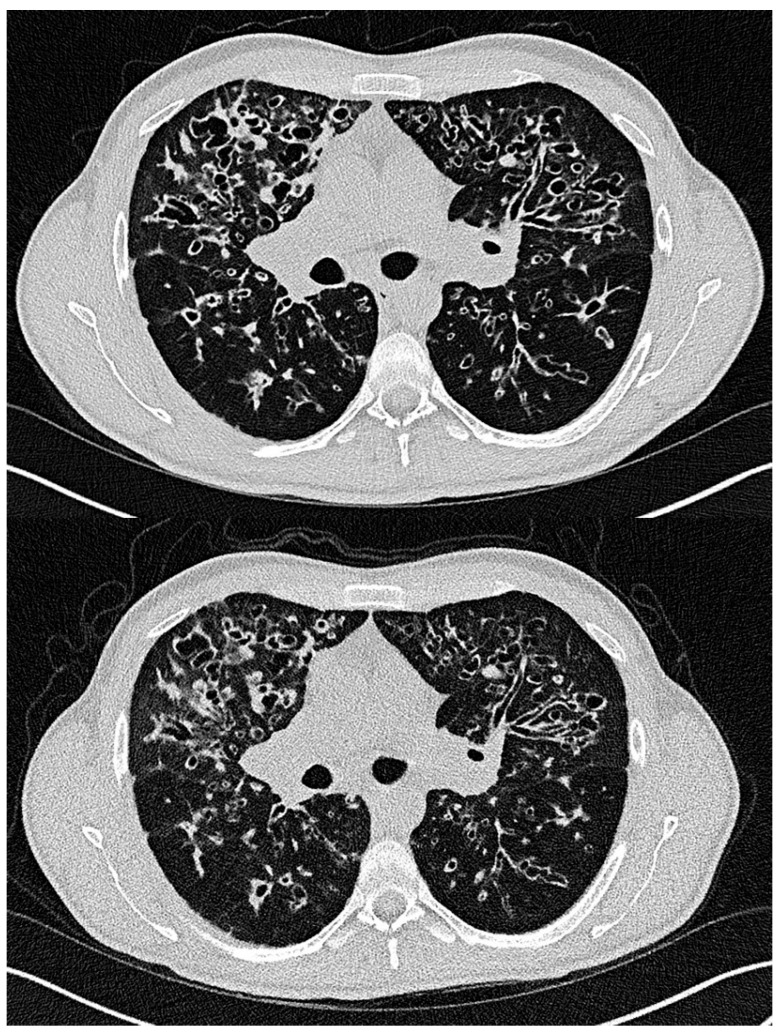
Exemplary illustration of the LD-HR (**top**) and ULD-HR (**bottom**) protocols in a patient with cystic fibrosis who received both protocols. The chest CT performed with the ULD-HR protocol was not included in the study. The ULD-HR image still allows the visualization of bronchial wall thickening, bronchiectasis and mucus plugging. LD-HR low-dose high-resolution, ULD-HR ultra-low-dose high-resolution.

Figure 3 shows examples of LD-HR and ULD-HR images from the same patient, highlighting the bronchial wall thickening, bronchiectasis and mucus plugging with overall good assessability. This 37-year-old patient was the only one examined with both protocols at different times during clinical routine (first scan July 2023 and second scan January 2024).

### 3.4. Radiation Dose

The tube current was significantly lower in ULD-HR scans than in LD-HR scans (ULD-HR: 45.95 [IQR: 18.25] mAs, LD-HR 133.65 [IQR: 35.91] mAs, percentage difference 66%, *p* < 0.001, Table 4). Consequently, the volumetric CT dose index, dose length product, and effective dose were also significantly lower in the ULD-HR images than in the LD-HR images (e.g., effective dose ULD-HR: 0.19 [IQR: 0.04] mSv, LD-HR: 0.55 [IQR: 0.08] mSv, percentage difference: 65%, *p* < 0.001, Table 4).

The organ doses showed similar results as well. The organ dose of the lung was 67% lower in ULD-HR scans compared with LD-HR scans (ULD-HR: 0.40 [IQR: 0.07] mSv, LD-HR: 1.22 [IQR: 0.20] mSv, *p* < 0.001, Table 4).

## 4. Discussion

This study aimed to evaluate a ULD-HR protocol in comparison to an LD-HR protocol for chest imaging using PCCT and to determine the reliability of the images at a reduced radiation dose. The dose in the ULD-HR protocol was reduced by 66% compared to the LD-HR protocol. Although the images from the LD-HR were rated slightly better overall, the image quality of the ULD-HR was predominantly good according to the Likert scale used in this study.

This study involved an interindividual comparison of two cohorts. Although slight differences were observed in mean age and BMI, and more pronounced variations in gender distribution, none of these parameters differed significantly between groups. Therefore, individual demographic variability is unlikely to have significantly influenced the effective dose. Regarding the assessability of bronchi, bronchial wall thickening, and bronchiolitis, some degree of subjective assessment bias cannot be ruled out. However, the outcomes of the quantitative evaluation characterized by increased image noise for the ULD images align with the marginally lower subjective ratings of the ULD images.

Several studies have examined the differences between LD-HR and ULD-HR protocols in terms of quantitative and qualitative image quality analyses. Tækker et al. compared the diagnostic value of LD and ULD protocols on EID-CT for detecting lung pathologies like bronchiectasis in a systematic literature review [21]. The sensitivity for bronchiectasis was 82–96% for LD protocols and 53–88% for ULD protocols. The ULD images exhibited lower sensitivity than the LD images. However, they remained at least partially diagnostically useful [21]. Similar results were obtained by Suliman et al. in a systematic literature review of LD and ULD chest CT protocols on EID-CT for imaging COVID-19 pneumonia [22]. The image quality is lower for ULD images with increased image noise, but is still at a diagnostically sufficient level [22]. Dettmer et al. investigated the diagnostic performance of a ULD chest CT protocol using PCCT compared with chest radiography [14]. Their study demonstrated a higher detection rate with the ULD protocol while maintaining diagnostic accuracy [14]. These findings are consistent with the existing literature. Although the reduction in radiation dose led to increased image noise and a lower signal-to-noise ratio compared with LD-HR protocols, the ULD-HR images remained diagnostically reliable (mean image quality and assessability of bronchi, bronchial wall thickening, and bronchiolitis were good) despite a slight decrease in subjective evaluability. This is also reflected in the slightly lower inter-rater reliability for the ULD-HR protocols compared to the LD-HR protocols with at least substantial agreement.

Several studies have examined ULD protocols using EID-CT in terms of radiation dose. In a systematic literature review, Suliman et al. reported effective dose values for ULD protocols ranging from 0.20 to 0.28 mSv [22]. Similarly, Greffier et al. found comparable results, with effective doses of 0.20 mSv in their study on pneumonia detection during the COVID-19 pandemic [23]. Wassipaul et al. compared ULD chest CT using EID-CT to chest X-ray, reporting an effective dose of 0.22 mSv for CT [24]. Building on this, Dettmer et al. compared ULD PCCT and chest X-ray for chest imaging and achieved an effective dose of 0.11 mSv for PCCT, although there were limitations in the assessment of the lung parenchyma [14]. A European guidance for ULD chest CT with EID-CT in children and adults with CF recommends effective doses of 0.08 mSv, whereby the image quality was not explicitly evaluated [5]. The results of this study, with an effective dose of 0.19 mSv, align with those of previous studies. Compared to Dettmer et al., the effective dose observed in this study was slightly higher, primarily due to the lower pitch value (1.5 vs. 2) and the higher image quality level (5 vs. 3) [14]. In relation to the diagnostic reference value for a two-plane chest X-ray examination of 0.1 mSv [25,26], the average effective dose in this study was only approximately twice as high.

Future research should focus on reducing the dose below that of a chest X-ray while accepting limitations in image quality. These limitations could be compensated through AI-driven image post-processing. Initial study results have shown a 75% dose reduction while preserving the diagnostic image quality [27].

Although current research findings and the potential for further dose represent an important step toward lowering the cumulative radiation dose in PwCF, the limited availability of PCCT and its higher cost compared to chest X-ray remain significant barriers to its widespread adoption in this patient population.

This study has some limitations. First, it was a retrospective single-center study, and its statistical impact could be enhanced by a prospective multicenter study design. Second, an intraindividual comparison would provide greater comparability. Third, according to the literature, an even greater dose reduction to the average value of a two-plane chest X-ray would have been possible. Fourth, the qualitative image quality analysis could achieve greater comparability through the use of evaluation scores such as Bhalla, Brody, or PRAGMA-CF.

## 5. Conclusions

In summary, the ULD-HR protocol on PCCT delivers only twice the effective dose of a two-plane chest X-ray for PwCF while maintaining good diagnostic image quality. This represents a significant advancement in CF patient care, as medical radiation exposure is an increasing concern in PwCF due to their improved life expectancy and high cumulative radiation dose. For future research and generalizability, multicenter or prospective studies would be desirable.

## Figures and Tables

**Table 1:** Patient characteristics.

Characteristics	LD-HR	ULD-HR	*p*-Value
Age, years	35.89 ± 12.48	33.19 ± 10.66	0.422
Female sex, n (%)	10 (27.78)	20 (55.56)	0.279
Male sex, n (%)	26 (72.22)	16 (44.44)	
BMI	21.83 ± 2.99	23.08 ± 4.66	0.236

LD-HR low-dose high-resolution, ULD-HR ultra-low-dose high-resolution; Values are mean ± standard deviation, or number of patients (%).

**Table 2:** Quantitative Image Quality Analysis.

	Region of Interest (Hounsfield Units)		|SNR|		
	LD-HR	ULD-HR	LD-HR	ULD-HR	*p*-value (SNR)
Lung	−910.72 ± 137.97	−905.61 ± 158.86	6.75 ± 1.21	5.88 ± 1.18	<0.001
Autochthonous back muscles	47.08 ± 157.64	48.92 ± 169.50	0.30 ± 0.09	0.30 ± 0.08	0.322
Air outside the patient	−1019.50 ± 99.33	−1024.00 ± 114.92	10.58 ± 2.03	9.30 ± 2.05	0.012

LD-HR low-dose high-resolution, ULD-HR ultra-low-dose high-resolution, SNR signal-to-noise ratio; Values are mean ± standard deviation (signal ± noise).

**Table 3:** Statistical Comparison of Image Quality Analysis.

				95% CI		Inter-Rater Reliability	
	LD-HR	ULD-HR	*p*-value	LD-HR	ULD-HR	LD-HR	ULD-HR
Image Quality	4 [1]	4 [0]	<0.001	[4.34–4.53]	[3.85–3.96]	0.85 [0.66–1.04]	0.67 [0.48–0.86]
Image Sharpness	4 [1]	4 [1]	<0.001	[4.33–4.52]	[3.48–3.67]	0.89 [0.70–1.07]	0.83 [0.65–1.02]
Image Noise	4 [0]	3 [0]	<0.001	[3.79–3.93]	[3.11–3.27]	0.77 [0.58–0.96]	0.73 [0.55–0.91]
Assessability of bronchi, bronchial wall thickening and bronchiolitis	4 [1]	4 [0]	<0.001	[4.35–4.55]	[3.73–3.90]	0.82 [0.63–1.00]	0.74 [0.56–0.91]
	LD-HR			ULD-HR			
	M.F.	H.T.	M.O.	M.F.	H.T.	M.O.	
Image Quality	4 [1]	4 [1]	4 [1]	4 [0]	4 [0]	4 [0]	
Image Sharpness	4 [1]	4 [1]	4 [1]	3.5 [1]	4 [1]	4 [1]	
Image Noise	4 [0]	4 [0]	4 [0]	3 [0.5]	3 [0]	3 [0]	
Assessability of bronchi, bronchial wall thickening and bronchiolitis	4 [1]	4 [1]	4 [1]	4 [0]	4 [0]	4 [0]	

CI confidence interval, LD-HR low-dose high-resolution, ULD-HR ultra-low-dose high-resolution. For ratings values are median [interquartile range], for inter-rater reliability values are Fleiss’ appa [95% CI].

**Table 4:** Comparison of Radiation Dose.

	LD-HR	ULD-HR	Difference	Percentage Difference	*p*-Value
CTDIvol (mGy)	0.78 [0.19]	0.26 [0.10]	0.52	67%	<0.001
DLP (mGy × cm)	26.37 [7.81]	8.43 [3.93]	17.94	68%	<0.001
Effective Dose (mSv)	0.55 [0.08]	0.19 [0.04]	0.36	65%	<0.001
Tube current (mAs)	133.65 [35.91]	45.95 [18.25]	87.70	66%	<0.001
Organ Dose (mSv)					
Lung (mSv)	1.22 [0.20]	0.40 [0.07]	0.82	67%	<0.001
Heart (mSv)	1.14 [0.24]	0.37 [0.07]	0.77	67%	<0.001
Skin (mSv)	0.39 [0.10]	0.14 [0.06]	0.24	63%	<0.001
Bones (mSv)	0.83 [0.18]	0.28 [0.07]	0.55	66%	<0.001
Esophagus (mSv)	0.73 [0.13]	0.25 [0.05]	0.49	66%	<0.001
Thymus (mSv)	1.31 [0.27]	0.42 [0.09]	0.90	68%	<0.001

CTDIvol volumetric CT-dose index, DLP dose length product, LD-HR low-dose high-resolution, ULD-HR ultra-low-dose high-resolution. Values are median [interquartile range], Difference = Value LD-HR − Value ULD-HR, Percentage Difference = Difference/Value LD-HR.

## Data Availability

The datasets generated and analyzed during the current study are not publicly available due to patient privacy regulations and institutional policies. Anonymized data supporting the findings of this study can be obtained from the corresponding author upon reasonable request and with approval of the local ethics committee.

## Abbreviations

CT	Computed tomography
CF	Cystic fibrosis
EID-CT	Energy-integrating detector CT
IQR	Interquartile range
LD-HR	Low-dose high-resolution
PCCT	Photon-counting CT
PwCF	People diagnosed with cystic fibrosis
ULD-HR	ultra-low-dose high-resolution
